# A Wearable Textile 2D Touchpad Sensor Based on Screen-Printing Technology

**DOI:** 10.3390/ma10121450

**Published:** 2017-12-20

**Authors:** Josue Ferri, Jose Vicente Lidón-Roger, Jorge Moreno, Gabriel Martinez, Eduardo Garcia-Breijo

**Affiliations:** 1 Textile Research Institute (AITEX), 03801 Alicante, Spain; josue.ferri@aitex.es (J.F.); jmoreno@aitex.es (J.M.); gmartinez@aitex.es (G.M.); 2 Instituto Interuniversitario de Investigación de Reconocimiento Molecular y Desarrollo Tecnológico (IDM), Universitat Politècnica de València, 46022 Valencia, Spain; jvlidon@eln.upv.es

**Keywords:** wearable sensing, touchpad, textile, screen-printing technology

## Abstract

Among many of the designs used in the detection of 2D gestures for portable technology, the touchpad is one of the most complex and with more functions to implement. Its development has undergone a great push due to its use in displays, but it is not widely used with other technologies. Its application on textiles could allow a wide range of applications in the field of medicine, sports, etc. Obtaining a flexible, robust touchpad with good response and low cost is one of the objectives of this work. A textile touchpad based on a diamond pattern design using screen printing technology has been developed. This technology is widely used in the textile industry and therefore does not require heavy investments. The developed prototypes were analyzed using a particular controller for projected capacitive technologies (pro-cap), which is the most used in gesture detection. Two different designs were used to obtain the best configuration, obtaining a good result in both cases.

## 1. Introduction

The flexibility and portability that wearables devices can bring have increased their development and their use. The interface devices, among all wearable devices, can be considered as indispensable tools for the user to interact with other devices both external and wearable [1]. Different strategies have been devised to integrate electronics as wearables such as smartwatches [2], rings [3], bracelets [4] as well as garments [5] identified as smart textiles or smart garments [6]. Each one of them has its advantages and limitations, being perhaps a complex garment, but, on the contrary, it is supposed to be more comfortable due to its comfort, flexibility, fashion, ergonomics and possibilities of integration in a transparent way [7,8].

An important factor to take into account in the use of wearable devices is the communication between the person and the electronic control system. This is carried out through the interfaces, such as keyboards, buttons and touchpads. The typical characteristics of these interfaces are the necessity of a stretchable or flexible setup to follow the action of human body [9] and a significant sensor area to detect the fingers [10].

Regarding touchpads, different technologies have been developed during the last years as solutions for “touch panel” and “touchscreen” [11]. From all of them, analog resistive and capacitive touch technologies dominate the touch landscape today [12]. Others have used optical sensing [13], force sensing [14], or even inductive sensing [15]. In combination uses, flexible and stretch sensing approaches have also been popular using contact resistances between threads [16,17], with piezoelectric [18] and capacitive designs [19,20].

Several designs and techniques have been used for touchpad application in textiles; in general, they can be classified into two main techniques: weaving the pattern by using fibers [21] or drawing the pattern on the fabric by using a printing techniques [22].

With regard to fibers, and depending on the sensor technology, different approaches of structures multilayer are developed. In the last years, Gu [20] reported a capacitor fiber highly flexible with a dielectric multilayer structure and conductive polymer composite films, manufactured by drawing techniques. Gorgutsa [23] built a fully woven 2D touchpad sensor and a 1D slide sensor manufactured with soft conductive-polymer-based capacitor. A flexible textile keyboard, using carbon nanotube (CNT) filled polypropylene (PP) composite fibers, is introduced [24] using conductive buttons made with threads combined with a metallic thread layer using a spacer material between both layers. The use of a textile polymer based conducting yarn and its characteristics are discussed in [21], giving details of the development of a touch control woven fabric keypad. A 7-bit dynamic range of pressure sensing at each taxel of piezoresistive multitouch arrays is achieved in [25] for musical applications using multitouch arrays sensing sandwiched structures with non-conductive pieces of fabric combined with parallel lines of conductive thread sewn into them. Hamdan [26] presented Grabrics, a two-dimensional textile sensor that is manipulated by grabbing a fold and moving it between fingers by using sewing techniques.

Regarding transferring a pattern on the textile, Takamatsu [1] presented a PEDOT:PSS based stretchable keyboard which is based on capacitance sensors; the electrodes are patterned on a knitted textile by using spin-coat technique. Dong-Ki Kim [27] reported a touchpad based on a contact-resistance-type force sensor manufactured by using screen-printing techniques.

In this article we will demonstrate a novel touchpad design fabricated over textile layer using the screen printing technique [28]. The well-known screen-printing technique is widely used in the graphic arts industry, ceramics, etcetera, and, in recent years, it has been introduced into the printed electronics industry. Its direct application in manufacturing allows reducing costs, while increasing profitability and reproducibility. Directly printing the touchpad on textile with this industrial technique would allow its easy production in series and its application in various areas.

## 2. Design and Working Principle

The working principle used is based on projected capacitive (pro-cap) technologies [29], which are commonly used for display systems but can also be used in other applications, as presented in this work. Pro-cap technologies detect touch by measuring the capacitance at each addressable electrode; in other words, when a finger approaches an electrode, the electromagnetic field is disturbed and alters the capacitance. The X, Y location, where the touch has occurred, can be located by measuring the variation of the capacitance with an electronic equipment.

There are two main types of pro-cap sensing methods, namely self-capacitance and mutual capacitance, each having its own advantages and disadvantages.

There are several pro-cap controllers which offer both self-capacitance and mutual-capacitance types. MTCH6102 from Microchip has been used in this work. This device is a turnkey projected capacitive touch controller that simplifies adding gestures to touch interface designs with industry-leading low-power performance. It utilizes up to 15 channels to support taps, swipes, and scrolling on XY touch pads and touch screens [30].

MTCH6102 has an embedded Capacitive Voltage Divider (CVD) acquisition engine which requires only an Analog-to-Digital Converter (ADC) to preform capacitive touch sensing. Capacitive Voltage Divider is a charge/voltage-based technique to measure relative capacitance on a pin using only the ADC module. The advantages of this technique are low power supply dependence, low-frequency noise rejection, low temperature dependence and minimal hardware requirements. The theory of operation can be found in Appendix A.

### 2.1. Sensor Pattern

Sensor pattern is a very important aspect of capacitive sensor design because the capacitance of touch is dependent on the sensor pattern design. Features, such as X-Y layer-to-layer spacing, electrode geometry and pitch, on front panel thickness and shielding must be considered when looking for the best design pattern. Attributes such as accuracy, resolution and linearity of touch position are greatly dependent on the sensor pattern as well.

The sensor pattern of touchpad commonly consists of a set of electrodes in a row and columns to form a matrix. Based on this structure, several touchpad–sensor pattern designs can be found, which are usually referred to by names that are indicative of the shape or construction of the pattern, such as telephone poles, snowflakes, triangles, diamonds, and streets and alleys.

**Figure 1:**
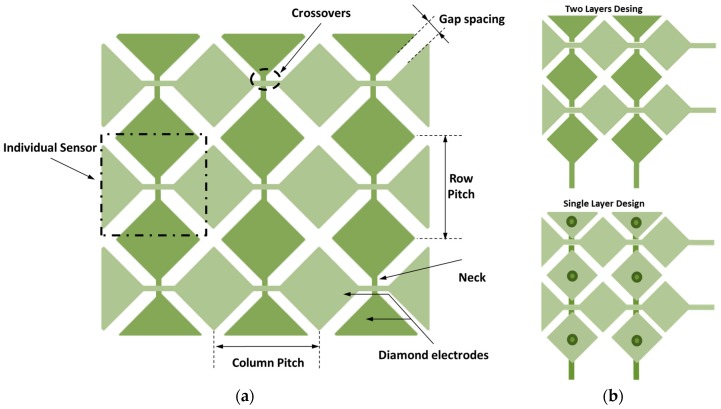
(**a**) Diamond pattern; and (**b**) 2D array sensors in the case of single layer or two layers.

Among the well-known pattern designs, the diamond pattern [31,32,33] is one of the most commonly used. It consists of diamonds interconnected with a narrow neck sections (Figure 1a).

The structure consists of two layers, each having a host of conductive electrodes organized parallel to each other. An individual sensors node is formed by the region between the edges of the X and Y electrodes (Figure 1a).

The diamond shape elements are used to maximize the exposure of sensor electrodes to a touch. There are two main parameters related to diamond pattern, namely pitch and gap. The pitch is the distance between the electrodes (Figure 1a) and its dimension determines the range of finger sizes that can reliably be detected. Typical dimensions [33] of the pitch are a minimum of 4 mm and a maximum of 10 mm. The gap between the X and Y electrodes (Figure 1a) defines how far a signal is projected and the level of noise in the measured signal as well. A sensor with a larger gap can detect a user further away, but it will have more noise than a sensor with a smaller gap. A minimum of 0.1 mm and maximum of 0.5 mm have been reported [33].

The Y electrodes are arranged among rows on the top layer and the X electrodes are arranged along columns on the top layer or bottom layer (Figure 1b), forming a two-dimensional array of sensors. The layers isolated from each other.

A ground ring around the touchpad can be placed to reduce the electromagnetic interference (EMI) to the active sensor area.

**Figure A3:**
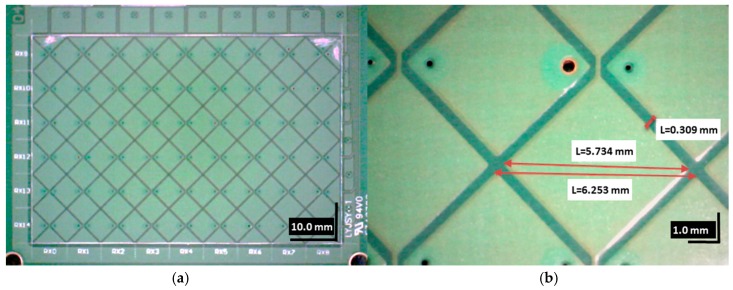
(**a**) One layer PCB 9 × 6 sensor matrix; and (**b**) gap and pitch size.

The low power projected capacitive touch pad development kit (Microchip DM160219) based on MTCH6102 Microchip device is an example of diamond pattern capacitive sensor, but manufactured with Printed Circuit Board (PCB) technology. In this case, a matrix of nine X-electrodes and six Y-electrodes is used (Figure A3). Pitch (Row and Column) of 6.2 mm and Gap of 0.3 mm are the dimensions of this design. The capacitance measure between electrodes is 20 pF (1 kHz) (Agilent U1731A LCR Meter, Santa Clara, CA, USA).

**Figure A4:**
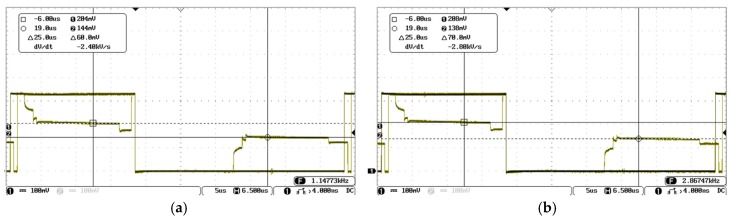
(**a**) “Released” signal; and (**b**) “pressed” signal.

The signal recorded on the RX0 line when there is no finger touching “released” (Figure A4a) shows a difference of 60 mV and, when there is finger touching “pressed” (Figure A4b), the difference is 70 mV.

### 2.2. Textile Touchpad Design

#### 2.2.1. Screen-Printed Technology

Manufacturing technology used to implement this type of sensor was based on serigraphic technology of thick film. The screen-printing process consists of forcing pastes of different characteristics over a substrate through some screens using squeegees. Openings in the screen define the pattern that will be printed on the substrate by serigraphy. The final thickness of the pastes can be adjusted by varying the thickness of the screens.

#### 2.2.2. Two Layers Design [TLD]

**Figure 2:**
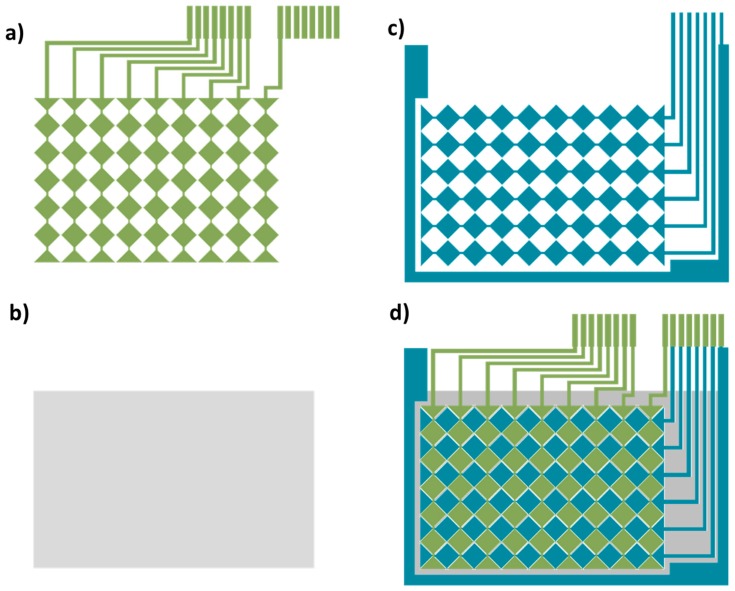
Two Layers Design (TLD): (**a**) Vertical or X layer; (**b**) dielectric layer; (**c**) Horizontal or Y layer; and (**d**) the complete design.

A sensor matrix formed by 9 × 6 electrodes has been designed. The sensor has been developed with two conductive layers for horizontal and vertical tracks and another layer of dielectric. The three patterns are shown in the Figure 2: Vertical or X layer (a); dielectric layer (b); Horizontal or Y layer (c); and the complete design (d).

The Pitch (Row and Column) of 8 mm and Gap of 0.4 mm are the main dimensions of pattern.

#### 2.2.3. One Layers Design [OLD]

**Figure 3:**
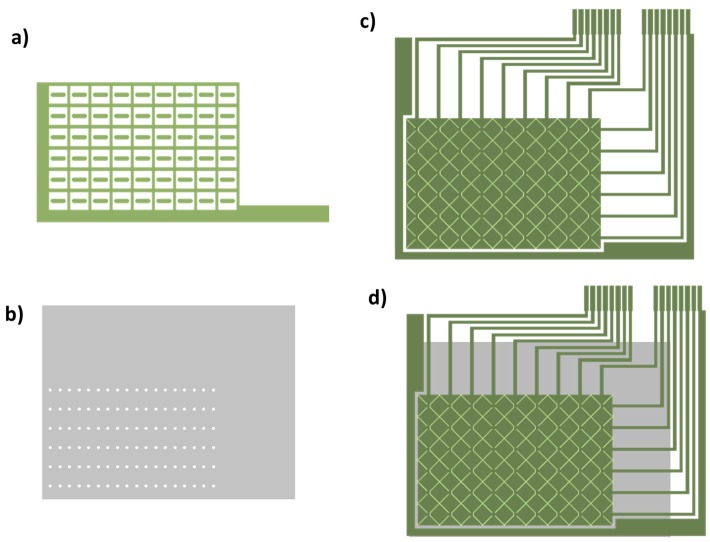
One Layer Design (OLD): (**a**) conductive layer for connection tracks; (**b**) dielectric layer with via-holes; (**c**) X-Y layer; and (**d**) the complete design.

A sensor matrix also formed by 9 × 6 electrodes has been designed with only one layer of horizontal-vertical (X-Y) conductive tracks. Despite being a one layer design, two extra layers are needed because the horizontal or vertical tracks have to be connected to avoid short circuits between them. Therefore, an extra conductive layer is added to make the connection and another extra dielectric layer is added to provide via-holes. The three patterns are shown in Figure 3: conductive layer for connection tracks (a); dielectric layer with via-holes (b); X-Y layer (c); and the complete design (d).

The Pitch (Row and Column) of 8.3 mm, Gap of 0.5 mm and through-hole diameter of 1.6 mm are the main dimensions of pattern.

### 2.3. Design of Electronic System

**Figure A5:**
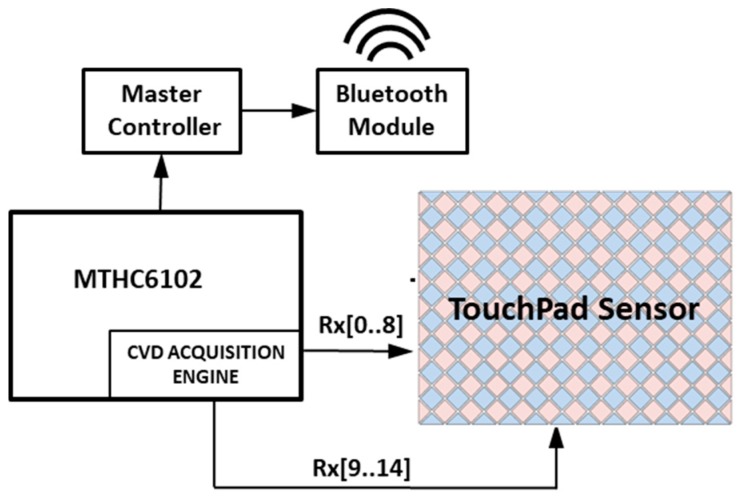
Electronic System Block Diagram.

The MTCH6102 low-power projected capacitive touch controller from Microchip was used to design the electronic system. As master controller a PIC16LF1454 was used. Finally, to make the system portable, a Bluetooth module was used (Figure A5).

## 3. Materials and Methods

### Sensor Development

When screen-printing technology is used, it is necessary to manufacture frames with screen mesh for each layer of the design. Therefore, to build the sensor matrices, three screens were made in both designs.

The screen for the conductors was a 230 mesh polyester material (PET 1500 90/230-48 from Sefar, Hong Kong, China) and the screen for dielectric layer was a 175 mesh polyester material (PET 1500 68/175-64 PW from Sefar). Afterwards, to transfer the stencil to screen mesh, a UV film Dirasol 132 (Fujifilm, Tokyo, Japan) was used. The final screen thickness was 10 μm for the screen for conductors and 15 μm for the screen for the dielectric. The patterns were transferred to the screen by using a UV light source unit.

The materials used were, the textile Mediatex TT ACQ 120 μm (Technohard, São Paulo, Brazil) for the substrate, C2131014D3 Silver paste-58, 85% (Gwent Group, Pontypool, UK) as conductive paste and D2081009D6 Polymer dielectric (Gwent Group) as dielectric paste. Flexibility is one of the most important characteristic of these inks to use them with textiles.

Printing was carried out by using Ekra E2 XL screen- printer with a 75° shore squeegee hardness, 3.5 bar force, and 8 mm/s. After inks depositing, these were cured in an air oven at 130 °C for 10 min.

**Figure 4:**
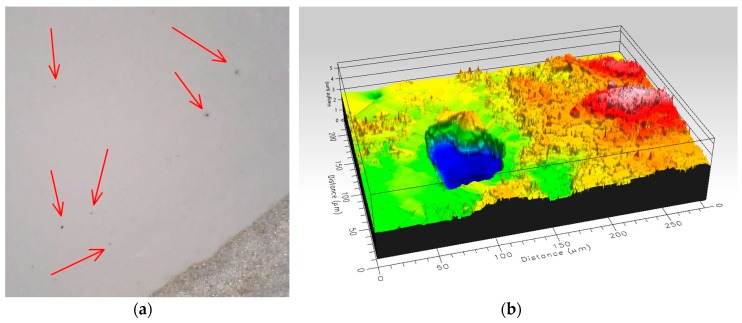
(**a**) Pinholes in the dielectric layer; and (**b**) detail of pinhole.

A high failure rate was found due to short-circuits between conductive layers. These short-circuits are due to pinholes in dielectric layer (Figure 4), which occur during the curing process.

To avoid this trouble, two solutions were found. The first solution was to increase the number of dielectric layers; however, this solution implies an increase in cost and processing time. Therefore, the ideal solution would be minimize the number of dielectric layers by using a different mesh screen size. For this reason, a study about the influence of dielectric layer mesh screen size was made. The mesh screen sizes selected were 123 inches (PET 1500 48/123-70 PW from Sefar), 137 inches (PET 1500 54/137-70 PW from Sefar), 175 inches (PET 1500 68/175-64 PW from Sefar), 230 inches (PET 1500 90/230-48 PW from Sefar) and 330 inches (PET 1500 130/330-34 PW from Sefar).

**Figure 5:**
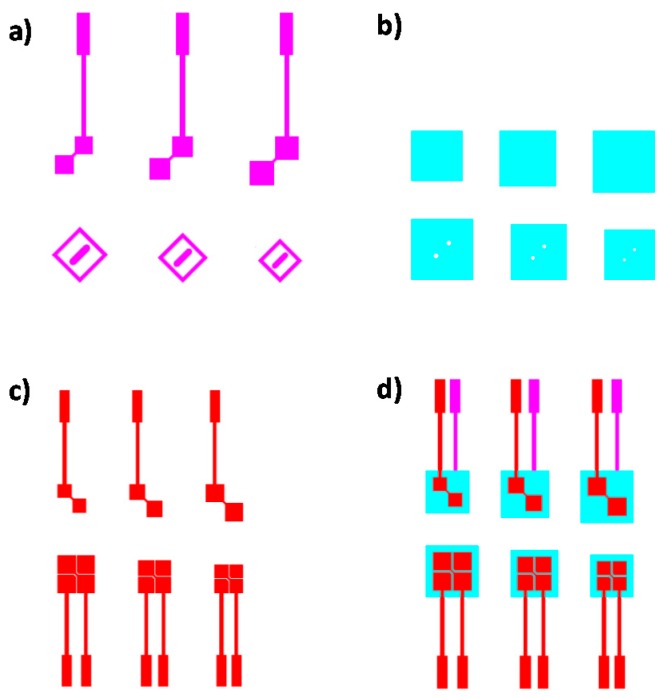
(**a**) First conductive layer, above for TLD and below for OLD; (**b**) dielectric layer; (**c**) second conductive layer; and (**d**) complete Design.

A single design pattern was designed (Figure 5) to study the influence of the mesh screen size for both cases, TLD and OLD. In both cases, three different single electrode sizes, which are designed called A, B and C; the conductive plates are square and their areas are 18, 24.5 and 32 mm^2^, respectively. Type C corresponds to the size of the electrodes used in the general design. The number of dielectric layers varied between one and three layers; to obtain the different layers, first one layer was printed and it was cured thermally; then the second one was printed and thermally cured; and, finally, the third one with the same process.

**Figure A6:**
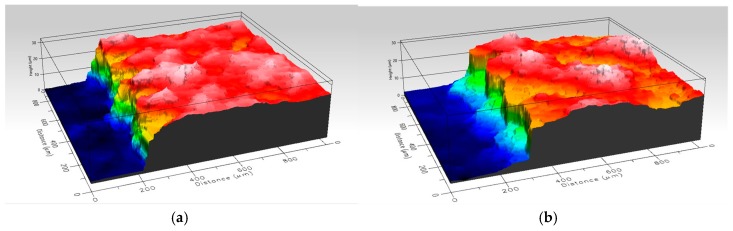
(**a**) Dielectric for a mesh of 175 and three layers; and (**b**) dielectric for a mesh of 137 and two layers.

Table 1 and Table 2 show the percentage of failures found. Table 3 shows the dielectric layer total thickness obtained for each mesh value and number of layers. For total layer thicknesses less than 10 μm, insulation faults are detected, as shown in Table 1 and Table 2. As a result, with a minimum of two layers, and using a mesh between 123 and 230, no errors are obtained in the manufacture of the devices. It is important to note that, as can be seen in Table 3, a similar layer thickness with two or three layers of dielectric can be obtained. For example, the thickness obtained with two layers and mesh 137 (Figure A6b) is similar to the thickness obtained with three layers and 175 mesh (Figure A6a). However, in the case of three layers, the surface obtained is more uniform than in the case of two layers, so a design with three layers would be more reliable than one with two layers. This may be because the ink is distributed through the different hollows left in the lower layers.

To look for dielectrics with different viscosities, a second solution was used. However, this change of viscosity adds a problem: the dielectric constant of the ink can change the value of the capacitance; therefore, it was necessary to study the influence of the dielectric on the value of the electrode capacitance. The ink D2081009D6 (Gwent) has a viscosity of 13.0–17.0 Pas (Haake VT550, PK1.1° at 230 s^−1^ at 25 °C) so a new dielectric ink was used, D2070209P6 (Gwent), which has a viscosity of 1.4–2.3 Pas (Haake VT550, PK1.1° at 230 s^−1^ at 25 °C).

The capacitances of the sensors were measured by using an inductance-capacitance-resistence (LCR) meter at 1 kHz (Agilent U1731A). Table 4 and Table 5 show capacitances for the dielectric D2081009D6. It is observed that there is very little variation (between 9 and 13) in capacitance in the case of the TLD design. However, in the OLD design, there is greater variation (between 13 and 60 pF) because, in this design, the influence of the variation of the surface of the electrodes and of their layer thickness is more noticeable. At higher mesh value, the layer thickness decreases; therefore, the capacitance increases, and the greater the surface of the electrode, the greater the capacitance.

The measurements for dielectric D2070209P6 show an increase in the capacitance of the electrodes (Table 6; to simplify, in this case, only the results are shown for a mesh of 175). The resulting capacitances are higher than those obtained with dielectric D2081009D6. Dielectric D2081009D6 was chosen for the final development of the touchpad since an increase of the capacitance supposes an incorrect operation of the controller.

The dielectric layer for the final designs was made with a screen of 175 mesh, using ink D2081009D6 and with two layers of dielectric in each prototype.

## 4. Results

### 4.1. Physical Parameters

**Figure 6:**
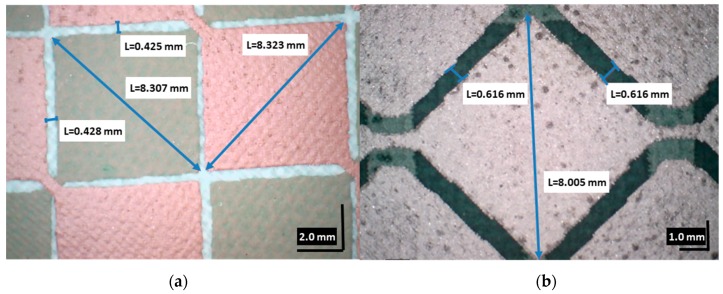
(**a**) Two-layer design; and (**b**) one-layer design magnification views.

The magnification view of the two designs with their dimensions are shown in Figure 6 (Figure 6a for TLD; and Figure 6b for OLD). The dimensions considered in the design have been obtained after manufacturing with an almost negligible error.

### 4.2. Electrical Parameters

**Figure 7:**
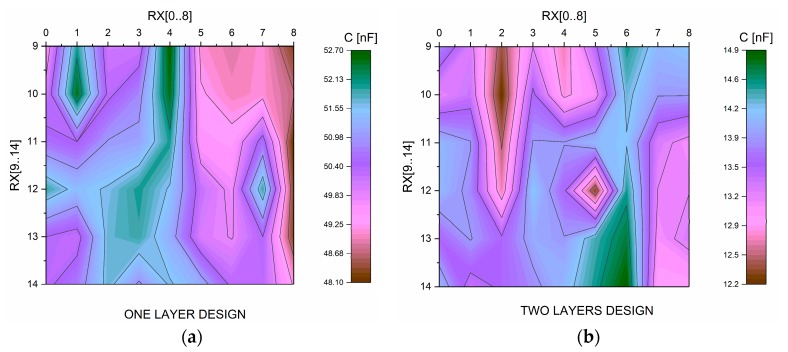
(**a**) Capacitance distribution on OLD; and (**b**) capacitance distribution on TLD.

The capacitance of the sensors has been measured using a RCL meter to 1 kHz (Agilent U1731A). Figure 7a,b shows the capacitance distribution in each sensor. The TDL has an average of 13 pF and OLD of 50 pF. In the case of the OLD design, a difference is observed over the value obtained in the simple electrodes previously performed, which may be due to the influence of the rest of the sensor array; this effect is not so noticeable in the case of TDL. In any case, they do not influence the correct operation of the touchpad.

**Figure A7:**
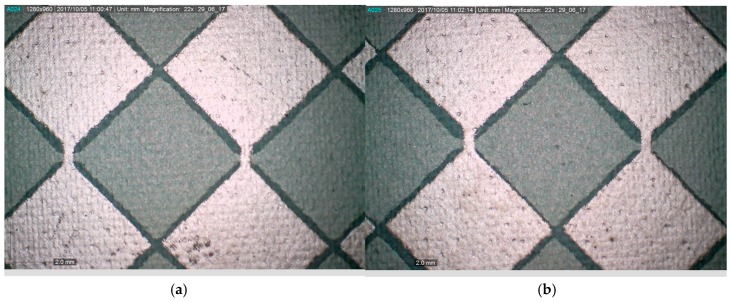
(**a**) Correct alignment; and (**b**) incorrect alignment.

Scattering observed in both cases is due to the fabrication process because the thickness of different layers is not exactly the same along substrate, and, in the case of OLD, it could also be due to an incorrect alignment between the layers which entails different geometries along the substrate (Figure A7).

**Figure 8:**
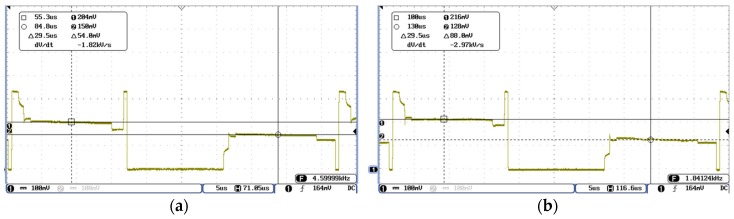
Two-layer design: (**a**) “released” signal; and (**b**) “pressed” signal.

With regard to TLD, the signal recorded on the R_XO_ line in “released case” (Figure 8a) shows a difference of 54 mV, while, in the “pressed case” (Figure 8b), it is 88 mV.

**Figure 9:**
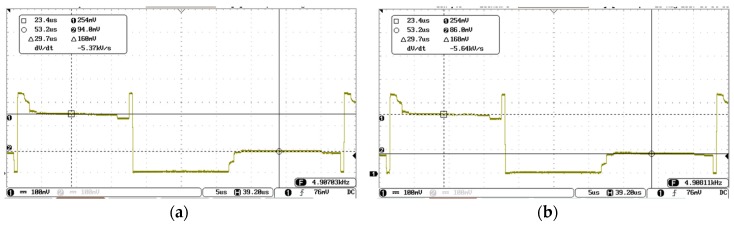
One-layer design: (**a**) “released” signal; and (**b**) “pressed” signal.

With respect to OLD, the signal recorded on the R_X0_ line in “released case” shows a difference of 160 mV (Figure 9a), while, in the “pressed case”, it is 168 mV (Figure 9b).

### 4.3. Design Using Different Types of Fabrics

Finally, after verifying the correct operation of the sensor, both TLD and OLD have been manufactured using the different types of fabrics shown in the Table A1.

**Figure 10:**
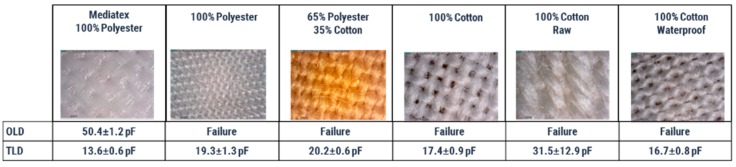
Capacitances and failures for each type of fabric and design.

Figure 10 shows the results for each type of fabric. In the case of OLD, all but Mediatex presented problems. The capacitance in this case is similar to that obtained previously. In TLD, there were no failures but the capacitance was not equal in all cases, with some dispersion of values in some cases such as that of 100% raw cotton. In the best cases, the capacitance was similar to that obtained previously.

The reason for failures and value dispersion may be due to loose threads, because they do not allow the dielectric to be an insulating layer. This is more notable in OLD as they have more conductive area faced.

### 4.4. Design Reducing the Size

**Figure 11:**
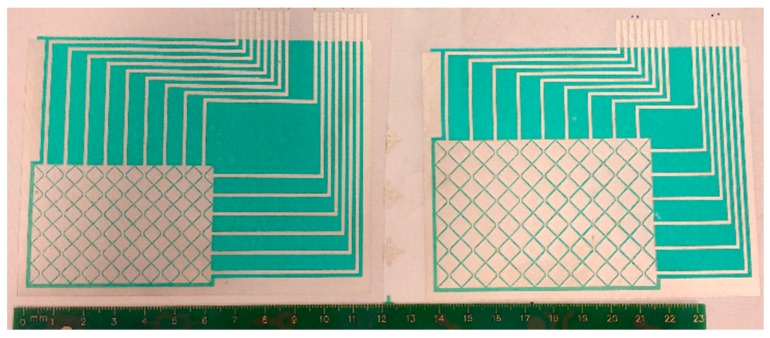
Touchpads with the same design but different size.

A new version of OLD was designed; the size of electrodes was reduced in this new version. The main dimensions of this new design pattern are: Pitch (Row and Column) of 6 mm, Gap of 0.5 mm and through-hole diameter of 1.2 mm (Figure 11). The prototype works properly and similarly to its larger version.

### 4.5. Operation

The MTCH6102 GUI utility allows checking all gesture detection of this device: Single Click, Click and Hold, Double Click, Right Swipe, Right Swipe and Hold, Left Swipe, Left Swipe and hold, Up Swipe, Up swipe and Hold, Down Swipe, and Down Swipe and Hold. Both designs were connected to the electronic system and tested with this GUI utility.

**Figure 12:**
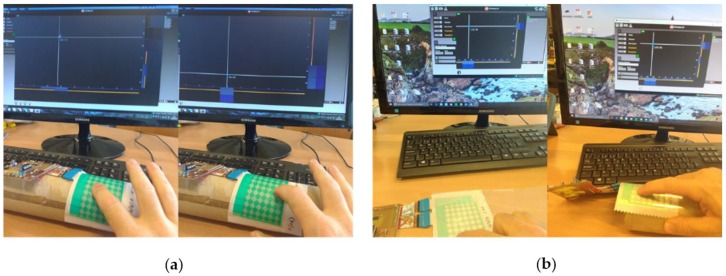
(**a**) Performance test on a curved surface touching two different points; and (**b**) performance test touching on the same point but on a flat and a curved surface, obtaining the same result.

In both designs, the operations worked correctly and all possible gestures could be checked; Figure 12a touching on a curved surface in two different points, and Figure 12b touching in the same point but on a flat surface (left) and on a curved surface (right), in both cases the resulting point is the same.

## 5. Conclusions

The capacitance obtained in both cases was similar to Microchip’s PCB design, but lower in the case of TLD. Capacitors in both cases have the same size; the difference lies in the dielectric: in TLD, there is a dielectric layer between the two conductive layers, whereas, in OLD, there is no dielectric layer. Despite the technology used, the distribution of capacitance along the sensors is quite uniform. Due to its low capacitance, the differential voltage obtained with TLD is higher than that obtained with OLD, as well as higher than Microchip’s PCB design. The initial failures found due to the manufacturing process have been solved.

A touchpad based on projected capacitive (pro-cap) technologies has been developed to be used with textile substrates using a low cost and habitual in the textile industry printing technique: screen-printing. The system works on both flat and curved surfaces, which allows it to be used in parts of clothes, such as sleeves, trouser legs or textiles for furniture such as sofas, armchairs, etc.

Touchpad design is based on a diamond type pattern. Two types of architecture with different sizes of electrodes and different types of textiles have been developed, and their correct operation has been verified. Being capacitive electrodes, the control of the capacitance is vital, which is why studies have been made on all those aspects that can modify the capacitance. The most important factor that conditions the capacitance is the thickness, thus it has been emphasized in the study of the factors that affect the thickness.

The application of this device to textiles depends on the type of textile surface, since it can modify the internal structure of the electrode; therefore, it is advisable to modify the surface of the fabric with a layer of dielectric.

## Figures and Tables

**Table 1:** Failures rate (%) for TLD when 1–3 layers of dielectric ink are used.

Mesh (Inch)	1 Layer			2 Layers			3 Layers		
	Type			Type			Type		
	A	B	C	A	B	C	A	B	C
330	100	100	50	0	100	0	0	100	0
230	50	0	50	0	0	0	0	0	0
175	0	0	0	0	0	0	0	0	0
137	0	0	0	0	0	0	0	0	0
123	0	0	0	0	0	0	0	0	0

**Table 2:** Failures rate (%) for OLD when 1–3 layers of dielectric ink are used.

Mesh (Inch)	1 Layer			2 Layers			3 Layers		
	Type			Type			Type		
	A	B	C	A	B	C	A	B	C
330	100	100	100	100	100	50	0	50	100
230	50	100	100	0	0	0	0	0	0
175	50	100	100	0	0	0	0	0	0
137	100	100	50	0	0	0	0	0	0
123	50	50	50	0	0	0	0	0	0

**Table 3:** Final layer thickness depending on mesh value and number of layers.

Mesh (Inch)	1 Layer	2 Layers	3 Layers
330	2.8 ± 0.6 μm	7.8 ± 0.3 μm	12.7 ± 1.1 μm
230	4.8 ± 2.1 μm	11.8 ± 0.8 μm	18.1 ± 1.2 μm
175	7.5 ± 2.4 μm	15.4 ± 1.2 μm	22.9 ± 1.5 μm
137	9.1 ± 1.1 μm	20.3 ± 1.4 μm	30.3 ± 0.9 μm
123	10.3 ± 0.2 μm	21.6 ± 2.1 μm	34.7 ± 2.3 μm

**Table 4:** Capacitance (pF) for TLD when 1–3 layers of D2081009D6 ink are used.

Mesh (Inch)	1 Layer			2 Layers			3 Layers		
	Type			Type			Type		
	A	B	C	A	B	C	A	B	C
330	-	-	6.79	6.1	-	6.47	5.89	-	7.02
230	7.54	6.74	7.33	6.81	6.56	6.87	6.43	6.01	7.05
175	6.72	6.28	6.24	11.8	12.5	13	5.82	5.67	5.97
137	6.91	6.75	6.99	5.6	5.26	5.58	5.4	5.2	5.8
123	5.21	5.61	6.37	5.85	6.73	6.09	5.35	4.46	5.35

**Table 5:** Capacitance (pF) for OLD when 1–3 layers of D2081009D6 ink are used.

Mesh (Inch)	1 Layer			2 Layers			3 Layers		
	Type			Type			Type		
	A	B	C	A	B	C	A	B	C
330	-	-	-	-	-	78	27.15	106	-
230	53.42	-	-	17.72	19.81	25.76	14.79	15.05	18.32
175	53.7	-	-	21.3	25.43	32.45	12.29	13.65	16.34
137	36	-	99	12.1	13.3	17.2	9.5	10.34	12.64
123	19.6	30.8	38.3	11.69	12.2	23	9.2	9.5	11

**Table 6:** Capacitance (pF) for OLD and TLD when 1–3 layers of D2070209P6 ink are used for mesh of 175 inches.

Type of Design	1 Layer			2 Layers			3 Layers		
	Type			Type			Type		
	A	B	C	A	B	C	A	B	C
**TLD**	-	10.9	15.1	9.13	7.71	9.44	9.1	7.64	9.1
**OLD**	-	-	-	80.6	94.1	121	57.5	66.8	85.8

**Figure A1:**
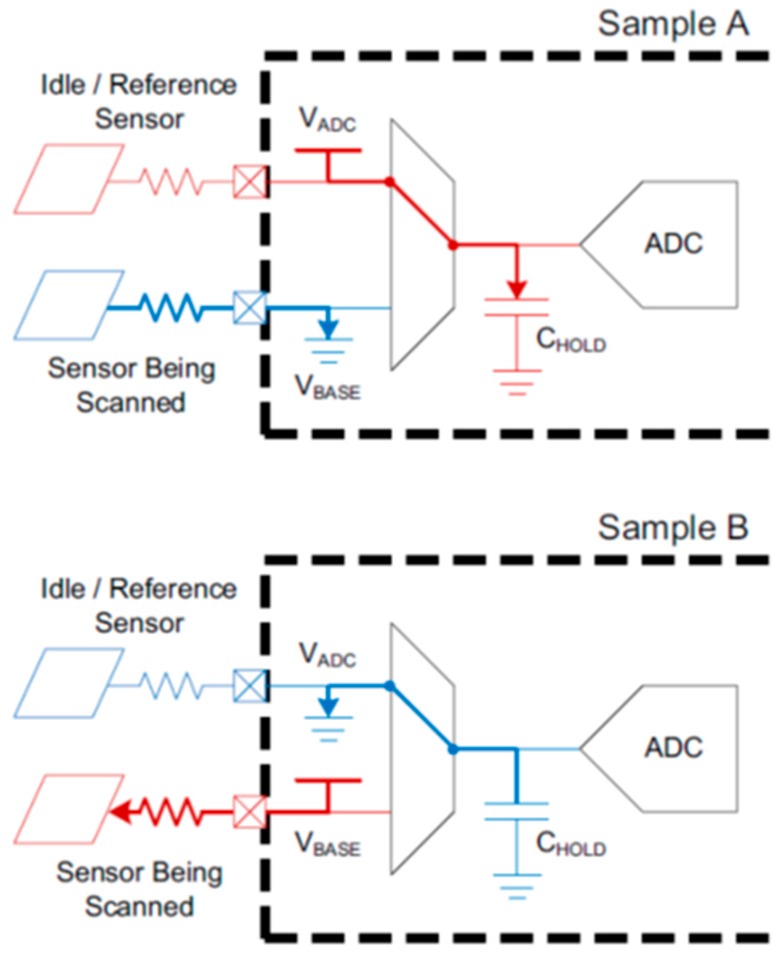
Precharge state: theory of operation [30].

**Figure A2:**
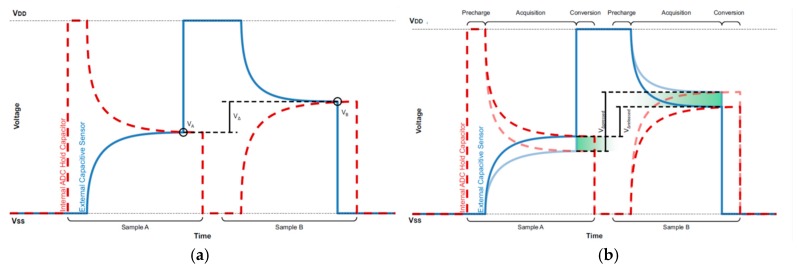
(**a**) Differential CVD waveform; and (**b**) difference between pressed and released differential values [30].

## Appendix A

The theory of operation can be explained based on Figure A1: in the first phase (Sample A), two capacitors are charged to different voltages; and, in the second phase (Sample B), the capacitors are charged again to opposite voltages. After each phase, the two capacitors are connected in parallel and the charges are allowed to settle until reaching the final voltage on C_hold_, which is determined by the size of the external capacitance in relation to the size of internal capacitance. The difference between the two voltages (V_B_-V_A_) is used as the currents sensor reading (Figure A2a). In the case of having a user’s finger capacitance, the evaluation is the same but with an extra capacitor (Figure A2b).

**Table A1:** Characteristics of the different fabrics used.

Fabric	Weft Density (Thread/cm)	Warp Density (Thread/cm)	Ligament	Grammage (g/m^2^)	Weft Material	Warp Material
**100% Cotton Waterproof**	34	45	Taffeta	120	Cotton	Cotton
**100% Cotton**	30	30	Taffeta	120	Cotton	Cotton
**100% Cotton Raw**	25	32	Satin	245	Cotton	Cotton
**65% Pol/35% Cot**	30	46	Taffeta	110	Cotton	Polyester
**Mediatex**	25	40	Taffeta	115	Polyester	Polyester
**100% Polyester**	28	40	Serge	120	Polyester	Polyester
